# Supplementary material for: The Expression of Toll-like Receptors in Cartilage Endplate Cells: A Role of Toll-like Receptor 2 in Pro-Inflammatory and Pro-Catabolic Gene Expression
Source: Cells. 2024 Aug 23;13(17):1402. doi: 10.3390/cells13171402 (PMC11394474; doi:10.3390/cells13171402)
Supplement: Supplementary file 1 [file cells-13-01402-s001.zip › Supplementary Figures_final.pdf]

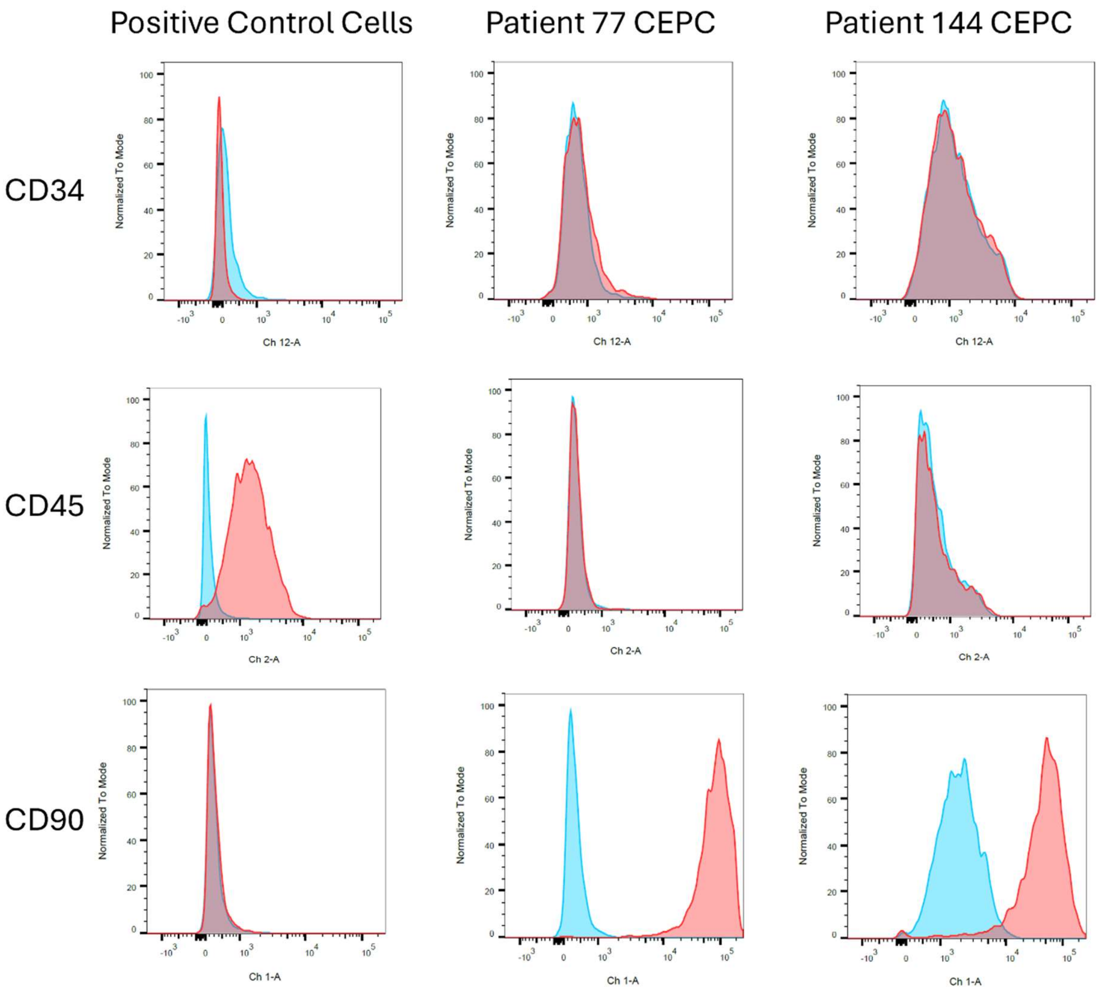

Supplementary Figure S1. The surface marker expression of CD34, CD45, and CD90 on CEPC was assessed. The left panels depict positive/negative control cells: THP1 cells for CD45+ and CD90- markers, and hematopoietic stem cells for CD34 positive expression. The middle and right panels show isolated CEPC from two patients at passages 1-2 exhibiting CD34-, CD45-, and CD90+ expression.

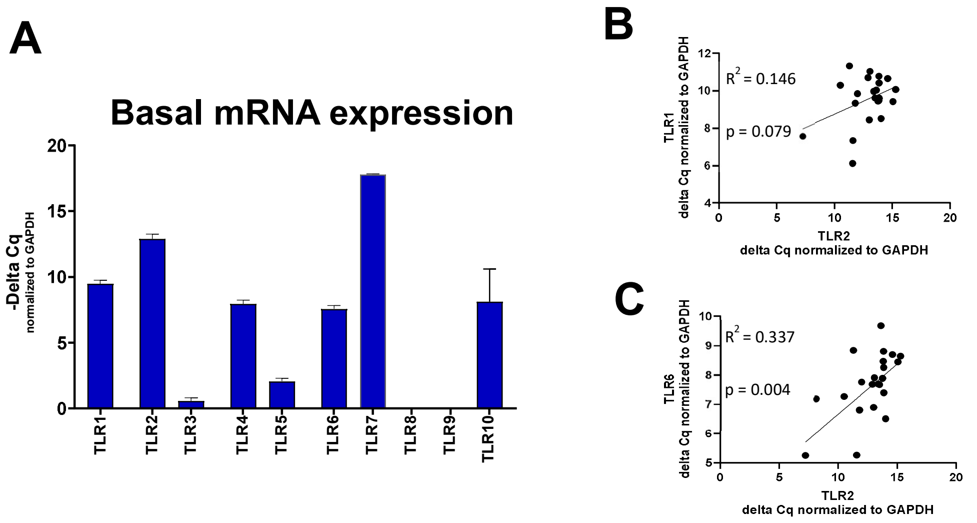

Supplementary Figure S2. (A) Basal TLR gene expression in CEPC presented as negative delta Cq values compared to reference gene GAPDH (mean GAPDH value = 24.91). **(B/C)** The correlation of TLR2 with **(A)** TLR1 and **(B)** TLR6, both of which it has the potential to form dimers with.
