# Supplementary material for: The Expression of Toll-like Receptors in Cartilage Endplate Cells: A Role of Toll-like Receptor 2 in Pro-Inflammatory and Pro-Catabolic Gene Expression
Source: Cells. 2024 Aug 23;13(17):1402. doi: 10.3390/cells13171402 (PMC11394474; doi:10.3390/cells13171402)
Supplement: Supplementary file 1 [file cells-13-01402-s001.zip › Supplementary Tables_.pdf]

Supplementary Table S1. Demographic characteristics of the patients from whom the cartilage endplates were obtained for measuring ex-vivo TLR expression. Surgical level, Pfirrmann grade of the adjacent disc, as well as age, gender, and body mass index (BMI) of the patients is documented.

Patient ID	Level	Pfirrmann Grade	Age	Gender	BMI
45	L4/5	3	69	Male	27.0
53	L5/S1	5	56	Female	27.1
61	L5/S1	5	67	Male	25.6
		Median = 5	Mean = 64	33.3 % Female	Mean = 26.6

Supplementary Table S2. Demographics of patients from which CEPs for immunohistochemistry stainings of TLR2 and TLR4 were derived. Level, age, gender and which TLR was stained for are documented.

Patient ID	Level	Age	Gender	Antibody
53	C4/5 and C5/6	53	Female	TLR2
48	C6/7	44	Female	TLR2
78	L5/S1	43	Female	TLR2
4	L4/5	70	Female	TLR4
57	C5/6 and C6/7	48	Female	TLR4
77	C6/7	51	Female	TLR4
		Mean = 51.5	100 % Female	

Supplementary Table S3. Demographic characteristics of patients from which nonMC and MC1 CEPs were derived. Surgical level, Pfirrmann grade of the adjacent disc, as well as age, gender, and body mass index (BMI) of the patients is documented.

		nonMC	MC1	P-value
**Number of patients**				
**Pfirrmann**		4.3 ± 0.8	4.0 ± 1.2	0.675
**Age**		57.4 ± 13.8	57.0 ± 18.1	0.959
**Weight (kilograms)**		81.3 ± 25.8	82.5 ± 23.0	0.926
**Height (cm)**		165.4 ± 12.2	173.9 ± 13.6	0.200
**BMI**		32.0 ± 8.0	26.7 ± 3.4	0.126
**VAS Back Pain**		6.1 ± 2.0	4.43 ± 3.74	0.454
**VAS Leg Pain**		5.6 ± 1.8	6.1 ± 2.3	0.541
**Oswestry Disability Score (ODI)**		30.5 ± 15.6	35.4 ± 24.8	0.617
**Females:Males**		05:05	03:04	>0.999
**Smoker (Yes : Ex : No)**				>0.999
	**Yes**	4	3	
	**No**	6	4	
**Level**				
	**L3/4**	2	1	

	**L4/5**	5	4	
	**L5/S1**	3	2	

Supplementary Table S4. Demographic characteristics of the patients from whom the cartilage endplate cells (CEPCs) were obtained for diverse in vitro stimulations using cytokines and TLR agonists. Surgical level, Pfirrmann grade of the adjacent disc, as well as age, gender, and body mass index (BMI) of the patients is documented.

Patient ID	Level	Pfirrmann Grade	Age	Gender	BMI
88	L4/5	5	77	Female	29.7
90	L5/S1	5	52	Female	30.3
92	L4/5	5	58	Male	50
93	L4/5	4	87	Male	25.9
97	L5/S1	3	43	Male	27
98	L3/4	5	84	Female	28.4
99	L3/4	4	60	Male	24.5
100	L4/5	3	64	Female	34.4
105	L4/5	2	49	Female	23.4
106	L4/5	4	39	Male	26.5
109	L4/5	3	36	Male	30.2
111	L5/S1	3	63	Female	21.3
112	L5/S1	4	54	Male	32.6
		Median = 3.8	Mean = 58.9	46 % Female	Mean = 29.6

Supplementary Table S5. Demographic characteristics of the patients from whom the cartilage endplate cells (CEPCs) were obtained for measuring TLR2 inhibition. Surgical level, Pfirrmann grade of the adjacent disc, as well as age, gender, and body mass index (BMI) of the patients is documented.

Patient ID	Level	Pfirrmann Grade	Age	Gender	BMI
44	L3/4	5	79	Female	22
58	L3/4	4	66	Female	30.9
77	L4/5	5	59	Male	32.4
82	L4/5	4	50	Male	28.5
101	L5/S1	2	73	Male	29.1
110	L5/S1	4	28	Female	29.4
112	L5/S1	4	54	Male	32.6
		Median = 4	Mean = 58.4	42.9 % Female	Mean = 29.3
